# SwarmRL: building the future of smart active systems

**DOI:** 10.1140/epje/s10189-025-00477-4

**Published:** 2025-04-07

**Authors:** Samuel Tovey, Christoph Lohrmann, Tobias Merkt, David Zimmer, Konstantin Nikolaou, Simon Koppenhöfer, Anna Bushmakina, Jonas Scheunemann, Christian Holm

**Affiliations:** Institute for Computational Physics, University of Stuttgart, Allmandring 3, 70569 Stuttgart, Baden-Württemberg Germany

## Abstract

This work introduces SwarmRL, a Python package designed to study intelligent active particles. SwarmRL provides an easy-to-use interface for developing models to control microscopic colloids using classical control and deep reinforcement learning approaches. These models may be deployed in simulations or real-world environments under a common framework. We explain the structure of the software and its key features and demonstrate how it can be used to accelerate research. With SwarmRL, we aim to streamline research into micro-robotic control while bridging the gap between experimental and simulation-driven sciences. SwarmRL is available open-source on GitHub at https://github.com/SwarmRL/SwarmRL.

**Supplementary Information:**

The online version contains supplementary material available at 10.1140/epje/s10189-025-00477-4.

## Introduction

Mastering control of microrobots at the microscopic scale has the potential for insights and the development of new technologies capable of changing the world. Whether it be an improved understanding of bacterial navigation strategies, emergent structure in active matter [1–4], or direct control over microscopic robots, numerous fields, including construction [5], plant pollination and ecosystem defence [6], and search and rescue [7], will be enhanced by advancements in microscale control research. One field in particular that will see significant changes is medicine, where it is expected that micro-robotic agents will be capable of advanced treatment strategies including targeted cancer therapies [8], drug delivery [9–12], and assisted fertilization [13, 14], amongst many others [15, 16]. The two most significant challenges in realising this future are developing the tools and materials to build such capable microscopic agents and programming them to achieve complex tasks with minimal observed input. These agents should not only be capable of performing complex actions such as swimming, pushing, and perhaps, communications, but they should do so without constant input from a supervisor. The first of these challenges has been, and is being, extensively investigated. Currently, there are many approaches for designing mobile microrobots [17]. Initial approaches included the light-driven Janus particles [18] and coils [19] and magnetically driven devices taking on numerous shapes [20, 21]. Recent approaches have also included using living cells to produce motion on this microscopic scale, the Xenobots being a prime example [22, 23]. However, the control of these agents, once built and deployed, is a complicated problem. Due to the size of these agents, directly installing computational processing power onto them is, thus far, intractable. Therefore, simplistic algorithms that aim to encode reactionary behaviour into the agents are often constructed, something that would require limited on-board processing. Examples of this include the use of microrobots to perform chemotaxis [24] by changing their shapes upon exposure to varying fields, object manipulation [25] through the use of an external magnetic field, and reproducing swarming behaviour by applying simple rotations and translations to individual agents by a set of rules triggered by changes in local environment [26, 27]. More recent approaches have utilized machine learning [28], oftentimes reinforcement learning, approaches to further push in the direction of learned control strategies [29]. Qin *et. al.* [30] utilized a Q-learning algorithm to learn swimming strategies in gated microswimmers, Borra *et. al.* [31] used an actor-critic framework to learn competitive capture-evasion strategies from hydrodynamic cues, and Muiños-Landin [32] investigated actor-critic learned navigation strategies under the influence of stochastic environments. In all cases, demonstrating under what conditions microrobots can perform complex tasks in various environments brings us a step closer to realising the full potential of this technology.

It is clear that research in the direction of micro-robotic control is both promising and excitingly multi-disciplinary; however, due to the nature of the technology required, whether it be complex experimental equipment, deep knowledge of state-of-the-art machine learning algorithms and their implementation, or the expertise to construct physically realistic simulations, the entry barrier can be high for specialists who focus on only one of these areas. For this reason, we have developed the SwarmRL Python package to combine each control workflow element under a common framework and accelerate algorithm development and deployment. SwarmRL enables researchers to apply control strategies to particles in simulation or experiments. These strategies can be built from sets of rules that are rigidly programmed and utilize the environment of the particles, or they can be trained using the actor-critic reinforcement learning approach, offering complete flexibility in how the agents are controlled, what actions they are capable of, and what tasks they must perform. Beyond the customizability, wherever possible, it is built on top of the JAX [33] ecosystem to maximize performance and is suited for deployment on large distributed compute clusters. This paper aims to introduce the SwarmRL software and demonstrate how it can be applied to perform frontier research. We begin with an overview of the theory underpinning the software, explicitly discussing microscale active matter, simulating it in a physically realistic manner, and the reinforcement learning we have built to control it. Afterwards, the architecture of the software is described, detailing how each piece of the software fits together and what can be done with them. Finally, we discuss some of the unique features of SwarmRL, including performance and visualization capabilities, before concluding with an outlook of the project. With SwarmRL, we hope to enable scientists from diverse backgrounds to contribute to and develop the field of micro-robotics research and realize the potential of this technology.

## Theory

SwarmRL harnesses tools from a range of fields, the largest of which are active matter simulation and reinforcement learning. In order to understand and better utilize the software infrastructure, it is important to also have an understanding of the theory it implements. In this section, we introduce the important theoretical concepts on which SwarmRL is built, before exploring the architecture of the package.

### Active matter

#### Biological and artificial active systems

Active matter refers to systems that consume energy from their surroundings and are thus internally driven out of equilibrium [34]. All biological and human-made machines fall under this term. However, in the context of this work, we focus on the branch of active systems that consists of small active particles that use energy to perform persistent, directed motion. This much more narrow definition covers plenty of examples in biology and engineering. Many bacteria like Escherichia coli [35], archaea like Thermoplasma volcanium [36], and small eukaryotes like Chlamydomonas reinhardtii [37] use molecular motors and organelles like archaella, flagella, or pili to self-propel. Artificial microswimmers can be made from colloidal particles that use, e.g. catalytic reactions [38], self-generated temperature gradients [39], or magnetic fields [40] to generate propulsion. In some more rare cases, the active particles can self-propel and self-steer, i.e. use torque to actively change their direction. For bacteria, tumbling [41, 42] is a well-known mechanism for change of direction. Some artificial microswimmers can also be steered by, e.g. change in laser focus [26] or magnetic field direction [43]. In all these examples, the active particle is of micrometre size and suspended in a liquid, making translational and rotational Brownian motion a non-negligible factor that competes with the deterministic active motion.

#### Simulation

Active matter systems with self-propelled small particles are usually modelled within the framework of stochastic dynamics of the individual constituents. We use the two- or three-dimensional overdamped Langevin equations

1 $$\begin{aligned}  &   \dot{{\textbf {r}}}_i(t) = \gamma _t^{-1} \left[ F^{\text {act}}_i(t) \hat{{\textbf {e}}}_i(t) + {\textbf {F}}_i({\textbf {r}}_i, \{{\textbf {r}}_j\}) \right] \nonumber \\  &   \qquad \quad + \sqrt{2 k_{\text {B}}T \gamma _t^{-1}} \varvec{\xi }^t_i(t), \end{aligned}$$

2where ${\textbf {r}}_i$ denotes the position of particle *i*, $\gamma _{t(r)}$ the translational (rotational) friction coefficient, $F^{\text {act}}$ the active driving force that models self-propulsion, $\hat{{\textbf {e}}}$ the unit vector representing the direction of the particle, ${\textbf {F}}_i({\textbf {r}}_i, \{{\textbf {r}}_j\})$ a conservative force from interactions between particle *i* and its environment as well as from interactions with other particles *j*, $k_{\text {B}}$ the Boltzmann constant, *T* the absolute temperature, $\varvec{\xi }^{t,(r)}$ the translational (rotational) noise with $\left\langle \varvec{\xi }^{t,(r)}_i(t)\right\rangle = 0$ and $\left\langle \varvec{\xi }^{t,(r)}_i(t)\otimes \varvec{\xi }^{t,(r)}_j(t')\right\rangle = \delta _{ij}\delta (t-t')\textbf{1}$, where $\left\langle \cdot \right\rangle$ denotes an ensemble average (expectation value) and $\textbf{1}$ the unity matrix, $M^{\text {act}}$ an active steering torque, $\hat{{\textbf {n}}}$ a unit vector perpendicular to $\hat{{\textbf {e}}}$ that sets the steering direction and ${\textbf {M}}_i({\textbf {r}}_i, \{{\textbf {r}}_j\})$ an interaction torque analogous to ${\textbf {F}}_i$. We stress the time dependence of $F^{\text {act}}$ and $M^{\text {act}}$ because SwarmRL is designed to focus on control and decision-making on the level of single particles. Collective behaviour and task fulfilment are to be achieved by the particles’ actions and what they can control rather than by external fields acting on all particles.

### Reinforcement learning

As a sub-field of ML, RL is concerned with how intelligent agents can take actions in an environment to maximize a cumulative reward. It allows these agents to learn optimal protocols to achieve a given task or a set of tasks without explicitly telling them how. This is oftentimes useful when a task is too complex for a simple algorithm or control system. It can also be used when the focus of the investigation is the emergence of a strategy for a problem. RL is, however, data-inefficient and is thus used mostly for problems where data exists abundantly and where the action space is relatively small such as autonomous driving [44], video games [45], and robotics [46]. It reached great popularity in 2016 when Deepmind’s AlphaGo won against the top Go player Lee Sedol [47]. When using it for specific tasks, however, RL can achieve remarkable results. In 2022, it solved a 50-year-old open question in mathematics how to most efficiently use matrix multiplication [48]; this discovery likely improves the efficiency of machine learning significantly due to its widespread use of matrix multiplication. It is further increasingly used in physics research for sophisticated tasks such as quantum error correction [49] and the magnetic control of tokamak plasmas [50]. This section provides an introduction to RL and explains the training approach currently implemented in SwarmRL, namely deep Actor-Critic (AC).

#### Actor-critic reinforcement learning

**Fig. 1:**
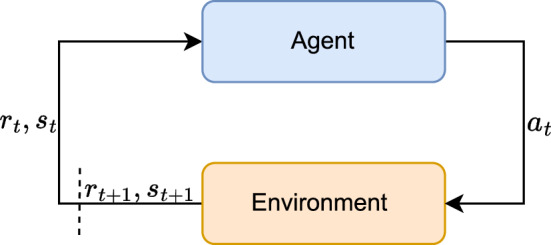
Graphical overview of the RL workflow. The agent selects an action, $a_{t}$, based in its current state, $s_{t}$, and acts within the environment. This action yields a new state, $s_{t+1}$, and an associated reward, $r_{t+1}$

The RL training paradigm is concerned with iteratively improving an agent’s decision-making such that it can achieve a pre-defined task as efficiently as possible. In practice, this is performed by placing agents into an environment with which they can interact by performing actions, $a_{t}$. To decide on such an action, the agent receives a state description, $s_{t}$, that describes the environment with sufficient information. The model used to decide on the action is referred to as the policy, $\pi _{\theta }: s_{t} \rightarrow a_{t}$, often taken to be a neural network with parameters, $\theta$ that is then referred to as an actor. These actors typically produce a probability distribution, which can then be sampled to select an action given the state. After an action is taken, a new state is produced along with a reward, quantifying the agent’s progress towards achieving its task, a process outlined in Fig. 1.

Fundamentally, the goal of training in RL is to find the optimal policy that, throughout a trajectory, $\tau$, maximizes a cost function, $J(\pi _{\theta })$, i.e.

3 $$\begin{aligned}  &   \pi ^{*} = \mathop {\mathrm {arg\,max}}\limits _{\pi } J(\pi _{\theta }) = \mathop {\mathrm {arg\,max}}\limits _{\pi } \int _{\tau }P\left( \tau | \pi _{\theta }\right) \cdot R(\tau ) \nonumber \\  &   \qquad = \mathop {\mathrm {arg\,max}}\limits _{\pi } \left\langle R(\tau ) \right\rangle _{\tau \sim \pi _{\theta }}, \end{aligned}$$

where $R(\tau )$ is the cumulative reward computed from a trajectory, $P(\tau | \pi _{\theta })$ is the probability of a trajectory given the current policy, and the angled brackets denote an average over many trajectories sampled under the $\pi _{\theta }$ policy. This is achieved by encouraging the policy to increase the probability of selecting actions that returned good rewards and discouraging those that didn’t. In practice, training is done throughout discrete-time episodes where state, policy probabilities, and reward data are collected and used to update the policy via the gradient ascent algorithm

4 $$\begin{aligned} \theta ^{'} = \theta + \eta \cdot \nabla _{\theta }J(\pi _{\theta }), \end{aligned}$$

with learning rate $\eta$, and

5 $$\begin{aligned} \nabla _{\theta }J = \left\langle \sum \limits _{t}^{T} \nabla _{\theta }\log \pi _{\theta }(a_{t}|s_{t})\cdot r_{t} \right\rangle _{\tau \sim \pi _{\theta }} \end{aligned}$$

with time step, *t*, and instantaneous reward, $r_{t}$. Typically, to ensure convergence in the long-term rewards and to encourage near-term over long-term progress, an expected returns function, $G_{t}$, will be used in the form

6 $$\begin{aligned} G_{t} = \sum \limits _{t^{'} = t}^{T}\gamma ^{t^{'} - t}r_{t^{'}}, \end{aligned}$$

where *T* is the final time step of the episode, and $\gamma$ is a discount factor set as a hyperparameter during training influencing the importance of late-time rewards. While this approach can be sufficient in training agents, it can break down in stochastic environments and high-dimensional spaces [51]. To address this, Sutton and Barto [52] showed that introducing a trainable baseline or value function, $V^{\pi }$, to compute an advantage function

7 $$\begin{aligned} A_{t}^{\pi } = G_{t} - V^{\pi }_{t}, \end{aligned}$$

can result in the stabilization of the expected returns and improved convergence time in the training, resulting in the reformulation of Eq. 5 to

8 $$\begin{aligned} \nabla _{\theta }J = \left\langle \sum \limits _{t}^{T} \nabla _{\theta }\log \pi _{\theta }(a_{t}|s_{t})\cdot A^{\pi }_{t} \right\rangle _{\tau \sim \pi _{\theta }}. \end{aligned}$$

When this value function is implemented as a neural network, it is often referred to as a critic, thus actor-critic reinforcement learning. The critic’s role is to determine the value of the state in which the agent currently finds itself. While several definitions exist, in SwarmRL, the value is defined as the theoretical returns possible if one starts in a state, $s_{t}$, and follows policy $\pi _{\theta }$. It improves this prediction by training on the true expected returns, Eq. 6, computed during episodes through a desired regression algorithm. Therefore, in Eq. 8, the advantage function guides training by encouraging selected actions that are better than or equal to those expected from the value function and discouraging those considered below what is theoretically possible. For this reason, AC RL training is often considered adversarial in nature, with the actor learning to beat the critic and the critic, in turn, learning the correct value of the encountered states. The training procedure outlined here is referred to as Vanilla Policy Gradient due to it being the simplest form of actor-critic training. More complex methods extend on the method discussed here, such as trust region policy optimization [53] and proximal policy optimization [54], the latter of which is also implemented in SwarmRL.

#### Multi-agent reinforcement learning

As SwarmRL specializes in controlling microscopic agents, there will be many occasions in which multiple agents must be simultaneously controlled and trained. This is addressed by multi-agent reinforcement learning (MARL) [55]. In MARL, *N* autonomous agents interact with the environment and each other. This extension brings several new aspects to the problem, such as cooperation and competition among agents and various information structures. In the following, we will address these aspects briefly.

***Cooperative, Competitive, and Mixed Setting***

In a fully cooperative setting, all agents share a common reward, i.e. $r^1=r^2=...=r^N = r$ or a team-average reward where $r=\frac{1}{N}\sum _{i\in N}r_i$ [56]. The latter is a generalized version of the former. Different reward functions for each agent allow more heterogeneity among agents [55]. A fully competitive setting in MARL is typically modelled as a zero-sum Markov game with $\sum _{i\in N}r^i=0$ [57]. Most literature focuses on two players where one agent’s reward is the other’s loss, something leveraged in, for example, predator–prey style games. A mixed setting imposes no restrictions on the goal and the relationship among agents [58].

***Information Structures***

Another critical challenge in MARL is the question *who knows what* during training and execution. This involves both the decision-making process and the optimization process. Three different forms of information structure are generally accepted. The first is the centralized setting with a so-called central controller aggregating joint observations, actions, and rewards and allowing for centralized learning. The execution, on the other hand, can either be centralized or decentralized. A centralized executed policy produces a joint action for all agents, while decentralized produces individually executed policies. The latter is the popular learning scheme of centralized-learning-decentralized-execution [59, 60]. On the other side, there is the fully decentralized setting in which each agent acts based only on local observation. This setting results in the so-called independent learning scheme [61]. A combination of both is a decentralized setting with networked agents [55]. This allows agents to share local information with their neighbours. The choice of setting depends on the problem at hand.

## Architecture

**Fig. 2:**
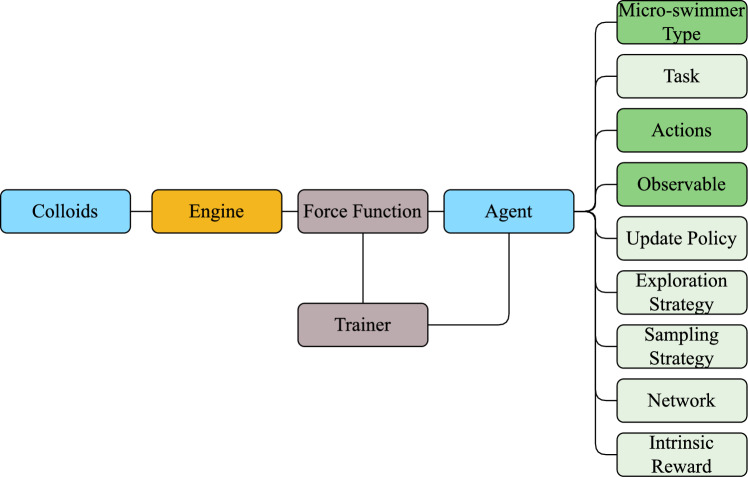
Overview of the SwarmRL software architecture. Light green boxes on the right-hand side of the figure represent modules with default settings that can be adjusted by users but do not need to be, dark green boxes indicate settings that need to be included in a system definition, blue boxes correspond to those directly handling colloid intelligence and properties, the engine is in orange, and grey represents classes that talk to the engine

SwarmRL has been designed to lower the entry barrier for scientists to access state-of-the-art reinforcement learning algorithms efficiently. It also offers a wide degree of customization and choice of algorithms that can be used during experiments to maximize the experienced user’s flexibility. To achieve both of these goals, SwarmRL is constructed using a modular, object oriented programming structure where almost all pipeline components are written as implementations of an abstract parent that defines the interface through which the components communicate. The Python programming language has been used throughout as it is most common amongst scientific disciplines; however, whenever possible, libraries have been used to improve performance through vectorized maps (vmaps), multi-threading, GPU use, and Just In Time (JIT) compiling [33]. A coarse-grained flowchart of SwarmRL is presented in Fig. 2 where each major component of the architecture is shown with its corresponding connections. The structure of SwarmRL is built around the interaction between the Engine, whether that be simulation or real experiment, and the Agents, the algorithms used to control the particles in the simulation. Connecting the Agents to the Engine is the Force Function. Force Functions are either constructed directly, or through a Trainer in the cases where trainable Agents are in use. Finally, the Agent holds Tasks, Actions, and Observables, each of which provide information and functionality to the algorithm being used. The rest of this section is dedicated to explaining each important component of SwarmRL, where they fit into the different capabilities of the software, and how they can be adapted or extended for individual implementations. It should be noted that, to reduce space and improve readability, all comments, type hints, and methods not directly related to the discussion have been removed from code samples. A full example application of SwarmRL to the study of chemotaxis using a variety of options is presented as an online Appendix.

### Engine

The Engine class of SwarmRL is one of its most important components. It represents the environment of the Agents and propagates the state according to the given actions. SwarmRL ships with an engine connected to the ESPResSo simulation software [62] to solve Equations (1) and (2) numerically. Through ESPResSo, we also offer the underdamped Langevin equation and a coupling to explicit hydrodynamics with lattice Boltzmann as the solver of the Navier–Stokes equations. SwarmRL also ships with rudimentary examples for connecting it directly to an experiment. Direct experimental connections are often kept private out of respect to the institutions that host the experiments. However, most implementations thus far use the TCP communications protocol to send actions and receive state information to hardware. Future work on SwarmRL aims to unify this communication procedure using the ROS ecosystem [63]. In cases where users must connect their own engines, be it simulation or experiment, they inherit from the Engine parent class. They must implement the two methods outline in Code Sample 1. 
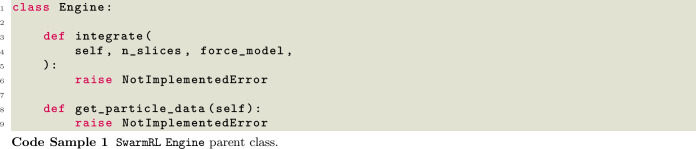
 The integrate method is used to step forward either in simulation or in an experiment. It takes as arguments the force model, which holds the control algorithms for the particles in the experiment, see Sect. 3.5, and number of slices to be looped over, that is, the number of times the force model is called for new actions to be applied to the particles. In a simulation, the time in-between successive time slices is filled with integrator steps, applying the chosen action at each time step. In contrast, in experiment, this would be applying the actions for a fixed period between slices. When instantiated, these parameters, time step or time between actions, are given to the specific engine. The second method that must be implemented is get_particle_data. This method must collect information about the particles in the system and give it back to the user uniformly so that all parts of SwarmRL can work with them. In its current state, SwarmRL defines colloids with properties outlined in Code Sample 2. 
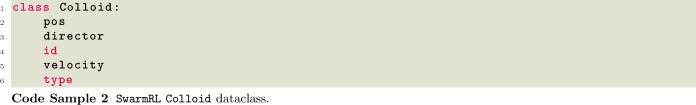
 Here, all information relevant to the models is assigned to a colloid class and used to predict new actions and to compute rewards.

### Agents

SwarmRL was built to service a variety of simulations and RL studies. A large component in enabling this is the Agent class, which manages how agents in the simulations are controlled, what they see, and what they are tasked with doing. The Agent collects the components introduced in the previous sections and represents the full set of sensing, control, and objectives of the colloids in question. This information can then be used by SwarmRL to effectively deploy these agents in the simulation environment. Code Samples 3 and 4 demonstrate the construction of two different agents which could both be added to a single simulation within SwarmRL.
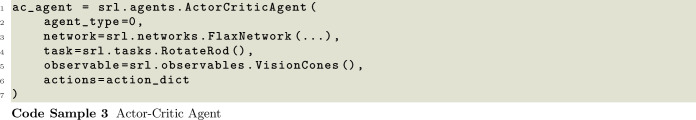

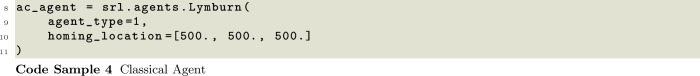


In the above examples, the actor-critic reinforcement learning approach controls one group of particles. This means their policy can be trained and adapted to new environments. The other example uses a so-called ClassicalAgent, in particular, one based on the swarming agents of [64].

### Classical control

SwarmRL allows users to control the actions of the particles in the system using both RL and classical algorithms. Classical algorithms, in this work, can be implemented without training and typically follow a defined set of rules. Such sets of rules have often been studied in the literature to reproduce behaviour in microswimmers [26, 27]. Classical agents are implemented directly into the Agent child class on the software side. However, they must follow the same structure; namely, at each time slice, the information of all particles in the system is passed into the class and in turn, it must return an action for each particle. In its current state, SwarmRL offers classical algorithms for the published models of Lymburn *et. al.* [64], Baeuerle *et. al.* [26], and Lavergne *et. al.* [27]. Future work aims at including model predictive control (MPC) algorithms into the SwarmRL ecosystem.

### Machine learning control

The machine learning side of agent control is driven by multi-agent reinforcement learning. However, due to the complex nature of this approach, several design decisions have been made to balance flexibility with limiting complexity. The main workhorse of the machine learning driven control is the Network class, which requires two methods to be implemented, compute_action which computes the action of the agent/s under study, and __call__, which returns action probabilities and, in the case of actor-critic approaches, also the predicted state values.

#### Architecture

SwarmRL currently utilizes the Flax neural network library [65] exclusively. In order to provide an interface for complex architectures, SwarmRL requires only a single Flax module to be passed to the FlaxNetwork class. This module must return both the action probabilities, and the predicted value function in case of actor-critic training. This approach allows implementing very complex neural network architectures such as graph models, with the same API interface as simple dense actors and critics. An example of two commonly used approaches is listed in Code Samples 5 and 6.
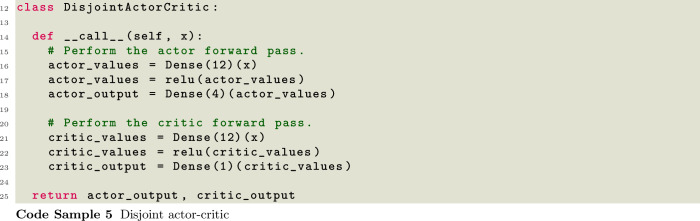

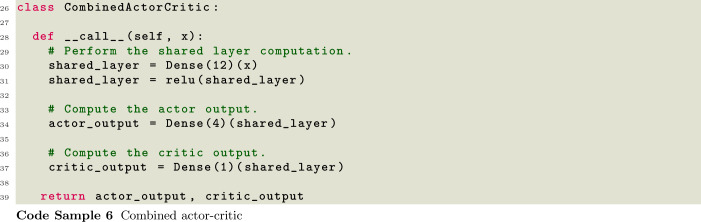


These code blocks show a disjoint and combined actor-critic approach bundled into the same Flax network structure. In this way, many complex neural network architectures can be constructed under a single module including graph or transformer modules.

Apart from the architecture, several components are essential to training AC models: the policy update algorithm, the approach used in the expectation computation, and the strategy for sampling actions from the learned distribution.

**Table 1:** Overview of RL algorithm options available in SwarmRL

Policy Update	Vanilla Gradient Update [66]	
	Proximal Policy Optimization [54]	
Returns	Expected Returns	
	Generalized Advantage Estimation [67]	
Sampling Strategy	Categorical Distribution	
	Gumbel Trick [68]	

#### Actions

Actions in SwarmRL are dictated by dataclasses which can be read by an engine and turned into forces, torques, or other values in a simulation or experiment. At the time of this report, SwarmRL has been focused on micro-robotics with standard direction control; therefore, the class is built from a simple set of attributes outlined in Code Sample 7. 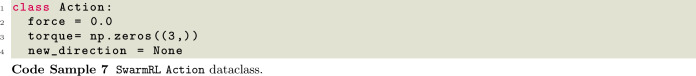 Force is applied along the agent’s director axis, torque describes the agent’s rotation, and new_direction provides a sometimes simpler approach to a torque calculation. From this template, we create an action by specifying the class’s force, torque, and new direction attributes and add them to a dictionary to be passed to an agent. For example, if the agent should be capable of translation forwards, translation backwards, clockwise, and counterclockwise rotation, the set of actions can be passed to the Agent as in Code Sample 8. 
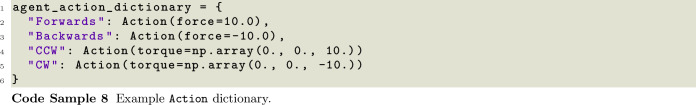


### Force function

The interaction model in SwarmRL is responsible for consuming data from the simulation and sending back computed actions for each of the agents. SwarmRL can control agents using machine learning and standard control theory approaches. The parent class for the models requires only a single function to be called by the Engine: calc_action, which takes a list of agent objects as input and returns a list of actions. This is the only occasion in which the environment communicates with the control algorithms and the communication must adhere to this very ‘narrow’ interface. This design choice enables novice SwarmRL users to switch out environments without adapting their RL setup and experienced users to easily implement new environments according to their needs.

Agent information is passed through as a dictionary when creating a model, demonstrated in Code Sample 9. 
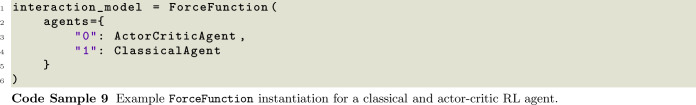
 In the case above, the experiment has at least two types of particles. One is controlled using an actor-critic reinforcement learning approach, while the other takes actions computed by a classical, non-trainable algorithm. In this way, SwarmRL users have complete control over the agents in their simulations and experiments.

### Tasks

As the goal of SwarmRL is to be used for the study of microscale robotic or biological systems to learn about their behaviour and control, it is often important to measure how well the system is doing. In RL, this is often referred to as the reward which is maximized during training. For classical algorithms, a task can also be used as a measure of how well the approach is doing or passed into the model as feedback. In SwarmRL, system performance measurement is handled by the Task class. The parent class of the task is built up of several methods that need to be implemented in the case of a custom task. The reduced class architecture is displayed in Code Sample 10. 
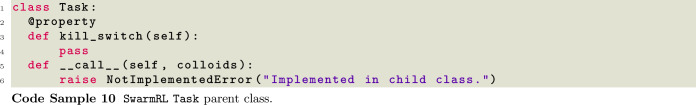
 The above class contains two fundamental components, the kill switch and the call method. As its name suggests, the kill switch is used to end the current run. If this property is set to True, SwarmRL will stop the current running engine and either restart it, or just finish. This is used, for example, in episodic training, where the training should stop and the engine should reset after an amount of time or when a criterion is reached. However, the kill switch can also be used for more critical cases, such as in an experiment when it appears that the agents would perform damaging actions. The second component, the call function, is used to compute a value describing the current state of this task; ideally, large positive values mean the task is being achieved, whereas negative values mean it is actively not being achieved. For example, when training RL agents to rotate an object, the call method would compute the rotation speed and return some positive value if it was increasing or large, and a negative value if it were decreasing.

At times, we want agents to perform more than just one task; in these cases, two options exist. The first is to write a custom task that provides feedback on two different objects such as how fast something is rotating and how far it has been pushed to a desired location. However, these tasks often quickly become convoluted and difficult to reuse. Therefore, SwarmRL provides the Multitasking class which takes a list of single tasks as an argument and applies them to the engine. This approach is more modular and allows for faster testing of strategies.

### Observables

A critical component of any RL algorithm and an important part of classical agent control approaches is their state description. Rather than passing all state information (e.g. all positions and directions of the agents) to the decision making model, the observable usually selects from or condenses the information considerably. This mimics the limited sensing capabilities that agents might have. For example, agents might only be able to retrieve information about other particles within a finite sensing radius or might not be able to tell the direction of their neighbours. Investigating the amount of information needed for an agent to still be able to perform its task is very relevant for the design of cost-efficient micro-agents. In SwarmRL, the description of the environment as sensed by the agents is computed by Observables. All observables implemented in SwarmRL inherit from a simple parent class outlined in Code Sample 11. 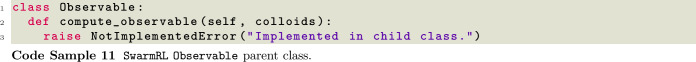 The class requires implementing one method, compute_observable, which takes a list of particle objects as input and returns the state description vector for each of them. This state vector is then passed on to the models by the force function. As with the Task class, we often want to construct agents capable of sensing the world in multiple ways. To achieve this, we also provide a MultiSensing class which takes a list of observables and computes a single output.

### Intrinsic reward

RL agents can be rewarded intrinsically to either encourage the exploration behaviour of an agent or to assist in building its knowledge about the environment [69]. As the name suggests, such a method is intrinsic to the agent and is therefore implemented as a separate sub-module IntrinsicReward which the agent can be equipped with.

#### Random network distillation

Random network distillation (RND) was introduced in 2018 by Burda *et. al.* [70] as an efficient method for exploring the state space of reinforcement learning agents. It has since been studied more broadly for its application and data selection for ML applications [71] and its implications on understanding data requirements for neural networks [72].

SwarmRL provides RNDReward, a module implementing RND, defining an intrinsic reward for agents, encouraging them to explore unseen regions of their state space. Going beyond the original version from Burda *et. al.* [70], SwarmRL provides two implementations of RND, differing in their capability to memorize previous states. For both versions of RND, a default setup is provided in . Utilizing such configuration the implementation of RND is shown in Code Sample 12. 
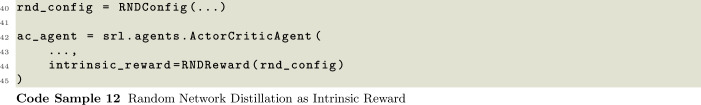
 The implementation of the RND backend is based on the ZnNL python package [73], offering a flexible but simple interface for complex training algorithms for neural networks.

### Exploration policy

In addition to the sampling strategy which can select actions from its distributions that do not have the largest probability, SwarmRL also provides direct access to exploration policies to further guide the agents into new, unexplored regions of their environment.

#### Random exploration

The simplest form of exploration policy is the RandomExploration class, which an exploration probability can parameterize, $\zeta$ and decay rate, $\epsilon$. The exploration rate dictates how likely it is for an agent to take an exploration action over a policy action, and the decay rate slowly decreases this probability as the training evolves by

9 $$\begin{aligned} \zeta ^{'} = \exp ^{-\epsilon \frac{t}{T}}\zeta , \end{aligned}$$

where *t* is simulation time, and *T* is the time for a single training episode.

### Trainer

The trainer is module in SwarmRL responsible for handling the updates of the models and their interaction with the environment while developing a policy. In the simplest case, the trainer deploys the models in the environment, collects the rewards over an episode, and performs a network update, as outlined in Code Sample 13. However, in many cases more is demanded during RL training and even how rewards are collected can change depending on the problem. Therefore, in SwarmRL, we have implemented several variations of the Trainer.

#### Continuous vs episodic

A user must first choose whether to perform episodic or continuous training. In episodic training, agents are deployed in the environment for a fixed number of steps or until some condition is met. After this time, a reward is computed based on their final state or trajectory during the episode. This reward is then used to update the networks before the environment is reset and the process is started again. In this approach, the episodes should be long enough to allow the agents to achieve their tasks but short enough to maximize efficiency. A similar but alternative approach is semi-episodic training, wherein the agents are updated continuously during the training but after some time, or again, once a condition is met, the environment is reset and the training continues. These options are available through the EpisodicTrainer packaged into SwarmRL. The EpisodicTrainer, displayed in Code Samples 13 and 14, allows users to define how often the environment should be reset as a function of RL-episodes, or to pass a task which can force an environment reset.
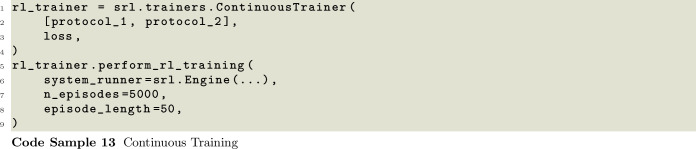

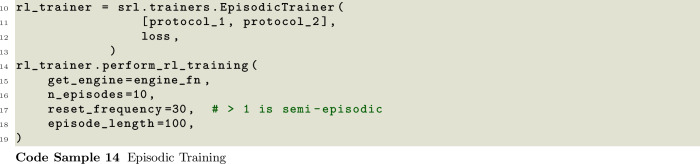


Continuous training is a paradigm in which agents are deployed into an environment and are updated after a fixed amount of time without resetting the environment. This online training is useful for situations where training must occur in the real world or where the time it will take to achieve a task is not well known. SwarmRL offers the ContinuousTrainer class to perform this kind of training. It should be noted that if a Task is provided to the continuous trainer capable of resetting the environment, the training will end. This can also be helpful in cases where an end to training can be strictly identified.

**Fig. 3:**
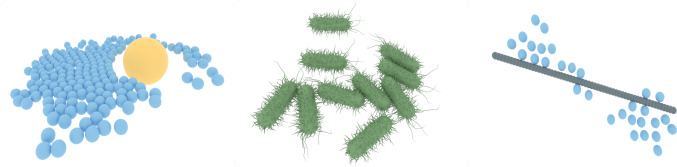
Example renderings using SwarmRL and the ZnVis visualization engine. (*left*) A snapshot from a reservoir computing experiment using a reinforcement learning controlled swarm. (*center*) Colloids are trained to perform chemotaxis in the same fashion as bacteria. Here, an open-source bacteria 3d model file has been used inside of SwarmRL to convey realism. The mesh was created by Sketchfab artist andrewfrueh, whose explicit permission was requested and granted before being used in this publication. The model is licensed under creative commons 4.0. (*right*) Demonstration of colloids rotating a rod, controlled using an RL algorithm

## Performance

Reinforcement learning typically does not require expensive neural networks, and classical control algorithms are limited in complexity by the number of particles they control. However, speed becomes critical when considering the number of iterations they have to go through during training and deployment. Therefore, SwarmRL has been built with a performance-first mentality, leveraging the JAX ecosystem [65] heavily. JAX is a Python library that, among other things, re-implements the NumPy library [74] with multi-threading, GPU deployment, and XLA-enhanced JIT compile capabilities. Most variables in SwarmRL are written using JAX arrays or their PyTree counterparts. PyTrees extend the functionality JAX has for NumPy objects to data structures like classes. Therefore, most of the class objects including the Colloid class can be placed onto a GPU or parallelized over. Furthermore, large parts of code are JIT compiled to ensure optimal performance.

## Software practices

In order to ensure the longevity and continued usability of the SwarmRL software, we adhere to several software practices. SwarmRL is thoroughly tested with unit, integration, and functional tests in place. Overall, we have 87 % coverage on the code with all classes having at least one unit test. The code follows the NumPy doc-string format and is extensively documented which is hosted at https://swarmrl.github.io/SwarmRL.ai/. Before new code is added to the project, all tests must pass and new tests should be included. The project is hosted on GitHub where all these standards are enforced automatically. Furthermore, we require at least one review from a code-owner, that is, a member of the core SwarmRL team, to accept new changes to the software.

## Visualization

Visualization is an essential part of the scientific process, particularly in simulations where one is searching for emergent strategy. It is not always clear from plots alone if the agents have learned what they should do. For this reason, SwarmRL interfaces closely with the ZnVis [75] visualization library. ZnVis, by design, handles the output data from SwarmRL and is developed alongside the project. The package can use custom mesh files for the agents, export rendered videos, and capture still-frame renderings using the Mitsuba engine [76]. Figure 3 illustrates the visualization capabilities of the package used on SwarmRL experiments.

## Conclusion

We have introduced SwarmRL, a Python package aimed at accelerating research into control and understanding of microscopic active particles, whether biological or artificial. SwarmRL allows researchers to implement state-of-the art control algorithms driven by reinforcement learning or classical policies, in both simulated and experiment settings. As a Python package, SwarmRL has a familiar interface for many scientists and is simple enough to use with limited background in software. Beyond its functionality, we have built SwarmRL to be performant, capable of running on many cores, GPUs, and scalable to HPC clusters. Our current focus is extending SwarmRL to handle more control algorithms, e.g. MPC, Q-learning, additional experiment implementations, and additional engines. Overall, our goal is that SwarmRL allows scientists to spend more time creating new ideas, building new technology, and changing the field, rather than implementing and staying up to date with state of-of-the art machine learning methods.

## Supplementary Information

Below is the link to the electronic supplementary material.

Supplementary file 1 (pdf 204 KB)

## Data Availability

The software package can be found publicly at https://github.com/SwarmRL/SwarmRL. No additional data were used in the study.
