# Supplementary material for: SwarmRL: building the future of smart active systems
Source: Eur Phys J E Soft Matter. 2025 Apr 7;48(4-5):16. doi: 10.1140/epje/s10189-025-00477-4 (PMC11976790; doi:10.1140/epje/s10189-025-00477-4)
Supplement: Supplementary file 1 — (pdf 204 KB) [file 10189_2025_477_MOESM1_ESM.pdf]

SwarmRL: Building the future of smart active systems

Samuel Tovey[1*†], Christoph Lohrmann[1*†], Tobias Merkt[1], David Zimmer[1], Konstantin Nikolaou[1], Simon Koppenhöfer[1], Anna Bushmakina[1], Jonas Scheunemann[1], Christian Holm[1]

[1*]Institute for Computational Physics, University of Stuttgart, Allmandring 3, Stuttgart, 70569, Baden-Württemberg, Germany.

*Corresponding author(s). E-mail(s): stovey@icp.uni-stuttgart.de; clohrmann@icp.uni-stuttgart.de;
Contributing authors: holm@icp.uni-stuttgart.de;
†These authors contributed equally to this work.

Keywords: Multi-Agent Reinforcement Learning, Microrobotics, ESPResSo MD, Artificial Intelligence, Active Matter

SwarmRL Example: Chemotaxis

Here we present an expanded use case of `SwarmRL`, studying the emergence of chemotaxis. This example was taken directly from the examples in the `SwarmRL` project.

```python
# SwarmRL Imports
import swarmrl as srl

# ESPResSo Imports
import espressomd

# Linalg Imports
import numpy as np

# Neural Network Imports
import flax.linen as nn
import optax

# Unit Handling
import pint

```

```python
# Plotting
import matplotlib.pyplot as plt

ureg = pint.UnitRegistry()  # Still define this outside.

system = espressomd.System(box_l=[1, 2, 3])  # This is just a dummy holder.
def get_system_runner(system):
    """
    Create a system runner.
    """
    md_params = srl.engine.espresso.MDParams(
            ureg=ureg,
            fluid_dyn_viscosity=ureg.Quantity(8.9e-4, "pascal * second"),
            WCA_epsilon=ureg.Quantity(293, "kelvin") * ureg.boltzmann_constant,
            temperature=ureg.Quantity(293, "kelvin"),
            box_length=ureg.Quantity(3 * [1000], "micrometer"),
            time_slice=ureg.Quantity(0.2, "second"),  # model timestep
            time_step=ureg.Quantity(0.02, "second") / 5,  # integrator timestep
            write_interval=ureg.Quantity(2, "second"),
        )
    system_runner = srl.engine.espresso.EspressoMD(
            md_params=md_params,
            n_dims=2,
            seed=np.random.randint(5453),  # seed for the simulation velocities
            out_folder="tutorial-2",
            write_chunk_size=1000,  # Used for dumping to the database.
            system=system,  # Add the pre-defined system.
        )

    # Add type 0 colloids to the simulation
    system_runner.add_colloids(
            n_colloids=20,  # Let's make 10 of them
            radius_colloid=ureg.Quantity(1.0, "micrometer"),
            random_placement_center=ureg.Quantity(
                np.array([500, 500, 0]), "micrometer"
            ),
            random_placement_radius=ureg.Quantity(60, "micrometer"),
            type_colloid=0,  # These are type 0
        )

    # Add type 1 colloids to the simulation
    system_runner.add_colloids(
            n_colloids=20,  # Let's make 10 of them
            radius_colloid=ureg.Quantity(1.0, "micrometer"),
            random_placement_center=ureg.Quantity(
                np.array([500, 500, 0]), "micrometer"
            ),
            random_placement_radius=ureg.Quantity(60, "micrometer"),
            type_colloid=1,  # These are type 1
        )

    return system_runner

def decay_fn(distance: np.ndarray):
    return 1.0 - distance

observable = srl.observables.ConcentrationField(
        source=np.array([500.0, 500.0, 0.0]),  # Source is the middle of the box
        decay_fn=decay_fn,
        scale_factor=1000,  # Scales the reward which might otherwise be very small.
        box_length=np.array([1000.0, 1000.0, 1000]),  # Normalizes distances.
        particle_type=0,  # Only acts on type 0 colloids.
    )

task = srl.tasks.searching.GradientSensing(
        source=np.array([500.0, 500.0, 0.0]),
        decay_function=decay_fn,
        reward_scale_factor=1000,
```

```
        box_length=np.array([1000.0, 1000.0, 1000]),
    )

actions = {
    "RotateClockwise": srl.actions.Action(torque=np.array([0.0, 0.0, 10.0])),
    "Translate": srl.actions.Action(force=10.0),
    "RotateCounterClockwise": srl.actions.Action(torque=np.array([0.0, 0.0, 10.0])),
    "DoNothing": srl.actions.Action(),
}

class ActorCriticNet(nn.Module):
    """A simple dense neural newtork."""

    @nn.compact
    def __call__(self, x):
        x = nn.Dense(features=12)(x)  # Shared layer,
        x = nn.relu(x)
        y = nn.Dense(features=1)(x)
        x = nn.Dense(features=4)(x)
        return x, y

network = srl.networks.FlaxModel(
    flax_model=ActorCriticNet(),
    optimizer=optax.adam(learning_rate=0.001),
    input_shape=(1,)  # Ipnut to the network is a single number.
)

agent = srl.agents.ActorCriticAgent(
    particle_type=0,
    network=network,
    task=task,
    observable=observable,
    actions=actions,
)

continuous_trainer = srl.trainers.ContinuousTrainer(
    [agent],
)

rewards = []

system_runner = get_system_runner(system)
rewards.append(continuous_trainer.perform_rl_training(
    system_runner=system_runner,
    n_episodes=500,
    episode_length=10,
))

episodic_trainer = srl.trainers.EpisodicTrainer(
    [agent],
)

rewards.append(episodic_trainer.perform_rl_training(
    get_engine=get_system_runner,
    n_episodes=500,
    system=system,
    episode_length=50,  # We should give them more time to reach the target.
    reset_frequency=1  # Increase this for semi-episodic training
))

semi_episodic_trainer = srl.trainers.EpisodicTrainer(
    [agent],
)

rewards.append(semi_episodic_trainer.perform_rl_training(
    get_engine=get_system_runner,
    n_episodes=500,
    system=system,
```

```python
    episode_length=10,
    reset_frequency=20  # Reset the environment after 20 episodes.
))
agent.save_agent("Models")

# Load the model and run it without training.
agent = srl.agents.ActorCriticAgent(
    particle_type=0,
    network=network,
    task=task,
    observable=observable,
    actions=actions,
)
agent.restore_agent("Models")

force_fn = srl.force_functions.ForceFunction({"0": agent})
system_runner = get_system_runner(system)
system_runner.integrate(1000, force_fn)
```

Code Sample 1 `SwarmRL` Chemotaxis Example.
